# EIN3 and ORE1 Accelerate Degreening during Ethylene-Mediated Leaf Senescence by Directly Activating Chlorophyll Catabolic Genes in *Arabidopsis*

**DOI:** 10.1371/journal.pgen.1005399

**Published:** 2015-07-28

**Authors:** Kai Qiu, Zhongpeng Li, Zhen Yang, Junyi Chen, Shouxin Wu, Xiaoyu Zhu, Shan Gao, Jiong Gao, Guodong Ren, Benke Kuai, Xin Zhou

**Affiliations:** State Key Laboratory of Genetic Engineering and Fudan Institute of Plant Biology, School of Life Sciences, Fudan University, Shanghai, China; Dartmouth College, UNITED STATES

## Abstract

Degreening, caused by chlorophyll degradation, is the most obvious symptom of senescing leaves. Chlorophyll degradation can be triggered by endogenous and environmental cues, and ethylene is one of the major inducers. ETHYLENE INSENSITIVE3 (EIN3) is a key transcription factor in the ethylene signaling pathway. It was previously reported that EIN3, *miR164*, and a NAC (NAM, ATAF, and CUC) transcription factor ORE1/NAC2 constitute a regulatory network mediating leaf senescence. However, how this network regulates chlorophyll degradation at molecular level is not yet elucidated. Here we report a feed-forward regulation of chlorophyll degradation that involves *EIN3*, *ORE1*, and chlorophyll catabolic genes (*CCGs*). Gene expression analysis showed that the induction of three major *CCGs*, *NYE1*, *NYC1* and *PAO*, by ethylene was largely repressed in *ein3 eil1* double mutant. Dual-luciferase assay revealed that EIN3 significantly enhanced the promoter activity of *NYE1*, *NYC1* and *PAO* in *Arabidopsis* protoplasts. Furthermore, Electrophoretic mobility shift assay (EMSA) indicated that EIN3 could directly bind to *NYE1*, *NYC1* and *PAO* promoters. These results reveal that EIN3 functions as a positive regulator of *CCG* expression during ethylene-mediated chlorophyll degradation. Interestingly, ORE1, a senescence regulator which is a downstream target of EIN3, could also activate the expression of *NYE1*, *NYC1* and *PAO* by directly binding to their promoters in EMSA and chromatin immunoprecipitation (ChIP) assays. In addition, EIN3 and ORE1 promoted *NYE1* and *NYC1* transcriptions in an additive manner. These results suggest that ORE1 is also involved in the direct regulation of *CCG* transcription. Moreover, ORE1 activated the expression of *ACS2*, a major ethylene biosynthesis gene, and subsequently promoted ethylene production. Collectively, our work reveals that EIN3, ORE1 and CCGs constitute a coherent feed-forward loop involving in the robust regulation of ethylene-mediated chlorophyll degradation during leaf senescence in *Arabidopsis*.

## Author Summary

Yellowing, caused by chlorophyll degradation, is the most obvious symptom of senescent leaves. Chlorophyll degradation can be triggered by a broad range of endogenous and environmental cues, and ethylene is one of the major inducers. Yet, the molecular regulation of chlorophyll degradation remains largely unknown. Here, we report a feed-forward regulation of ethylene-mediated chlorophyll degradation that involves ETHYLENE INSENSITIVE3 (EIN3), ORE1/NAC2, and major chlorophyll catabolic genes. EIN3, a master positive regulator of ethylene signaling, could directly promote chlorophyll degradation by physically binding to the promoters of three major chlorophyll catabolic genes to activate their expressions. Meanwhile, ORE1, a direct target of EIN3, also activates the expression of the similar set of chlorophyll catabolic genes directly. Moreover, ORE1 activates the expression of a major ethylene biosynthesis gene *ACS2* during senescence, and subsequently activates a positive feedback to ethylene synthesis. Our work reveals a feed-forward loop that promotes ethylene-mediated chlorophyll degradation during leaf senescence, advancing our understanding on the molecular mechanism of leaf yellowing.

## Introduction

Leaf senescence occurs at the final stage of leaf development and involves a series of changes at the molecular, cellular and phenotypic levels. Senescence is initiated by characteristic degenerative processes, e.g. chlorophyll (chl) degradation and macromolecule breakdown, and particularly recycling of nutrients to actively growing tissues or storage organs [1]. Molecular and genetic studies of *Arabidopsis thaliana* have identified dozens of senescence-related mutants and hundreds of senescence-associated genes (SAGs) involved in light signaling, hormone signaling and chl catabolism [2–4]. The phenotypic change of senescing leaves is degreening due to the net loss of chl in chloroplasts.

A biochemical pathway of chl degradation was recently elucidated in *Arabidopsis* via the identification of chl catabolic genes (CCGs). As the initial step, chl *b* is converted into chl *a* through two reductive reactions that are catalyzed by chl *b* reductase (NYC1/NOL) and 7-hydroxymethyl chl *a* reductase (HCAR), respectively [5–7]. Then the Mg atom of chl *a* is removed by an enzyme not yet identified to form pheophytin *a*. The phytol tail of pheophytin *a* is subsequently removed by pheophytin pheophorbide hydrolase (PPH) to produce pheophorbide *a* [8,9]. The ring structure of this intermediate product is then oxygenolytically opened by pheophorbide *a* oxygenase (PAO) to generate red chlcatabolite (RCC), which is degraded further by RCC reductase (RCCR) [10]. The conversion of pheophorbide a to RCC leads to the loss of green color during chl catabolism. Recently, these major chl catabolic enzymes (CCEs) were found to physically interact with STAY-GREEN1 (SGR1, also known as NYE1), a general regulator of chl degradation [11]. SGR1/NYE1 is essential for recruiting CCEs onto thylakoid membranes in senescing chloroplasts to promote chl degradation [11,12].

Plant hormones have been extensively reported to regulate leaf senescence, with ethylene, abscisic acid, jasmonic acid, brassinosteroid and salicylic acid functioning as inducers and auxin, cytokinin, and gibberellic acid as inhibitors [1,13–16]. Ethylene has long been considered a key endogenous regulator of leaf senescence and fruit ripening. Exogenous ethylene treatment accelerates leaf senescence, and the ethylene biosynthetic genes *1-Aminocyclopropane-1-carboxylic acid* (ACC) *synthase* (*ACS*) and *ACC oxidase* (*ACO*), were upregulated in senescing leaves, coupled with an increased ethylene level [15]. An octuple mutant of *ACSs* showed a stay-green phenotype [17]. In the absence of ethylene, ethylene receptors are in an activated form and activate a Raf-like kinase CONSTITUTIVE TRIPLE RESPONSE1 (CTR1), and CTR1 in turn suppresses the downstream ethylene response pathway. Ethylene binding results in the inactivation of the receptor function [18], leading to the deactivation of CTR1, which then releases the suppression of the downstream positive regulators ETHYLENE INSENSITIVE2 (EIN2) and EIN3 [19,20]. EIN2 is a central positive regulator of ethylene signaling that locates in the membrane of the endoplasmic reticulum [21], where it undergoes cleavage and nucleus translocation controlled by CTR1-directed phosphorylation [22–24]. EIN3 is the key transcription factor of ethylene signaling downstream of EIN2. The ethylene-insensitive mutant of the ethylene receptor gene *ETR1*, *etr1-1*, and loss-of-function mutants *ein2* (also known as *ore2*) and *ein3* were all isolated as delayed-senescence mutants [25–27], which suggests a key role of ethylene in the initiation and/or progression of leaf senescence.

The pivotal role of ethylene in leaf senescence was further confirmed by the revelation of a feed-forward loop, whereby EIN2 affects leaf senescence in part by regulating the expression of *miR164* and one of *miR164* target genes, *ORE1/NAC2* [28]. *ORE1/NAC2* is a member of NAC transcription factor family which has been shown to play an important role in leaf senescence. In particular, ORE1 is a positive regulator of leaf senescence, promoting the expression of senescence-associated genes *BFN1*, *SAG29*, and *SINA1* by directly binding to their promoters [29,30]. EIN3 was later on found to be involved in this feed-forward regulation by directly suppressing the expression of *miR164*, which negatively regulates *ORE1* at the post-transcriptional level [27]. Meanwhile, EIN3 also directly activates the expression of *ORE1* by binding to its promoter to accelerate leaf senescence [31]. These findings improve our understanding of the transcriptional regulatory cascade of ethylene signaling during leaf senescence and suggest that EIN3 positively regulates leaf senescence by inducing the expression of *ORE1* directly and indirectly. Although both mutants of EIN3 and ORE1 showed delayed senescence or stay-green phenotypes, the molecular mechanism of how these genes regulate chl degradation during senescence is still largely unknown.

In this study, we report that EIN3 promotes chl degradation via the direct up-regulation of major chl catabolic genes, *NYE1*, *NYC1* and *PAO*, by binding to their promoters. Meanwhile, one of the EIN3 direct targets, *ORE1/NAC2* [31], also directly activates the expression of *NYE1*, *NYC1* and *PAO* as well as *NOL*. Moreover, EIN3 and ORE1 promote *NYE1* and *NYC1* transcriptions in an additive manner. Intriguingly, ORE1 can also promote the transcription of *ACS2* for a positive feedback regulation of ethylene biosynthesis and signaling.

## Results

### EIN3 upregulates the transcription of *ORE1*

**Fig 1:**
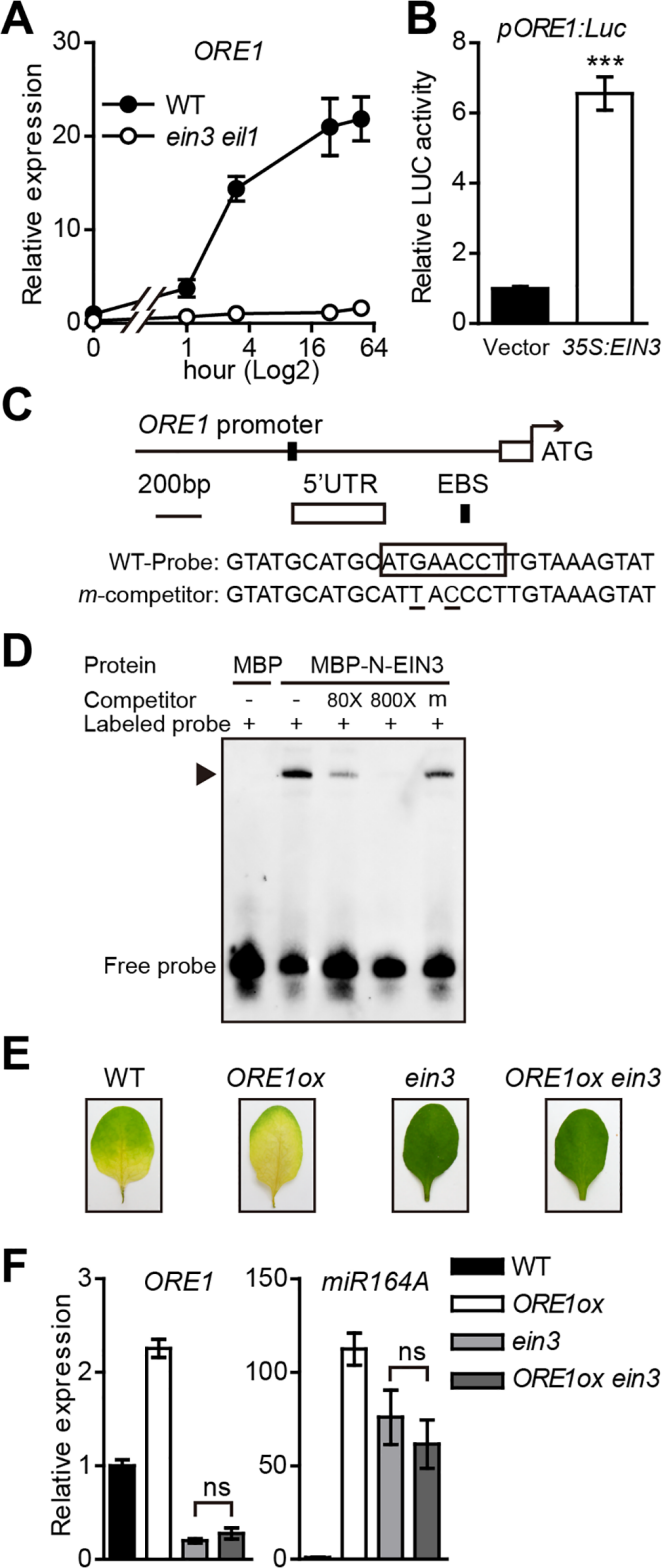
*ORE1* is directly activated by EIN3 and repressed by *miR164*. (A) Kinetic analysis of *ORE1* expression in leaves of the wild-type (WT) and *ein3 eil1* in response to ethylene treatment. Detached third and fourth rosette leaves from 4-week-old plants were treated with 100 μL/L ethylene for various periods of times. RT-qPCR was performed to quantify the *ORE1* mRNA levels. *ACT2* was used as an internal control to normalize different samples. The mRNA level of *ORE1* in the WT at 0 hr was arbitrarily set to 1. The x axis is shown in log2 scale. Data are mean ± SEM of 3 biological replicates with technical duplicates for each. (B) Transient dual-luciferase transactivation of the *ORE1* promoter by EIN3 in protoplasts from *Arabidopsis* leaves. Protoplasts were co-transformed with the *pORE1*:*Luc* reporter (1694 bp upstream from the translation start site of *ORE1*) and an effector overexpressing EIN3 (*35S*:*EIN3*). The *35S*:*REN* was serving as an internal control. Relative reporter activity was normalized by the ratio of LUC/REN. An empty vector was used as a negative effector control, with LUC/REN ratio arbitrarily set to 1. Data are mean ± SEM of 3 biological replicates. *** *p* < 0.001 (*t*-test). (C) Schematic diagram of putative EIN3 binding site (EBS) in the *ORE1* promoter. A 28-bp DNA fragment containing the EBS in *ORE1* promoter was used as the probe for EMSA. The putative EBS in the WT probe sequence is boxed. The consensus nucleotides of EBS in the competitor sequence (underlined) were mutated. (D) EIN3 proteins physically interact with *ORE1* promoter in EMSA. The N-terminus of EIN3 protein (aa 1–314 containing DNA binding domain) fused to maltose binding protein (MBP) was used to detect interaction (MBP-N-EIN3). MBP protein was used as a negative control. Biotin-labeled probes were added to each reaction mixture. WT competitor DNA was added in 80-fold and 800-fold molar excess. Mutated version of competitor DNA (m) was added in 800-fold molar excess. “‒” and “+” represent absence or presence, respectively. Triangle indicates the DNA-protein complex. (E) Representative leaves of WT, *ORE1ox*, *ein3*, and *ORE1ox ein3* plants subjected to ethylene treatment for 3 days. (F) qRT-PCR analysis of relative gene expression of *ORE1* and *miR164A* in leaves in (E). *ACT2* was used as reference gene. Expression of each gene in the WT was set to 1. Data are mean ± SEM of 2 biological replicates. ns: not significant.

EIN3 directly activates the expression of *ORE1/NAC2*, a master senescence-associated NAC transcription factor with significantly increased expression in the senescing leaves [27,28,31]. Our qPCR analysis confirmed that ethylene-induced *ORE1* expression was abolished in the leaves of 4-week-old *ein3 eil1* plants (Fig 1A), and the *35S* promoter-driven expression of *EIN3* significantly increased the activity of *ORE1* promoter in *Arabidopsis* mesophyll protoplasts (Fig 1B). These results are consistent with previously published data [31].

Although the association of EIN3 protein with the *ORE1* promoter was first revealed in Y1H and ChIP assays [31], the evidence of direct binding was not clear. We scanned the *ORE1* promoter and found a putative EIN3 binding site (EBS), ATGAACCT, located 1056~1064 bp upstream from the start codon (ATG) of the gene (Fig 1C). Electrophoretic mobility shift assay (EMSA) was used to determine the *in vitro* binding of EIN3 to its putative binding site with a recombinant truncated EIN3 that fused to MBP. This truncated version of EIN3 contains the first 314 amino acids of the N-terminus that covers the full-length DNA binding domain [32,33]. The MBP-N-EIN3 fusion protein could bind to the biotin-labeled wild-type (WT) probe (Fig 1D). Excessive amount of unlabeled competitor effectively competed with the binding, and the competition was dose-dependent: the binding signal was completely abolished with a sufficient amount of unlabeled competitor. Furthermore, once the putative EIN3 binding site was mutated, the unlabeled mutated probe could no longer compete for the binding. These results suggest that EIN3 positively regulates *ORE1* expression by directly binding to the EBS on its promoter during ethylene-mediated leaf senescence in *Arabidopsis*.

We then tested whether *EIN3* mutation affects the function of ORE1 protein. The *ein3* single mutant displayed a stay-green phenotype with ethylene treatment partly because it may lack a functional ORE1 protein. As expected, overexpression of *ORE1* in a WT background (*ORE1ox*) caused early leaf yellowing (Fig 1E). To check the effect of loss-of-function of *EIN3* on the ORE1 protein function, we crossed the *ORE1ox* line with the *ein3* mutant and examined the phenotype of *ORE1ox ein3* with ethylene treatment. The *ORE1ox ein3* line still exhibited a stay-green phenotype, which mimicked the phenotype of the *ein3* mutant (Fig 1E). The *ORE1* transcript was high in the *ORE1ox* line but very low in the *ein3* and *ORE1ox ein3* lines (Fig 1F). Considering the fact that these are the same transgenes in different genetic backgrounds, we reasoned that the *ORE1* transcript in *ORE1ox ein3* line was probably degraded. A previous study suggested that *ORE1* mRNA is a target of *miR164*-mediated cleavage [28]. The *miR164* family in *Arabidopsis* represses the expression of a group of *NAC* genes including *ORE1*. In addition, another recent paper reported that EIN3 negatively regulated *miR164* transcription by directly binding to its promoter [27]. So the high level of *miR164* in the *ein3* background may have degraded the *ORE1* transcript in *ORE1ox ein3*. Therefore, we checked the transcript level of *miR164A*, one of the 3 isoforms of the *miR164* family, and found the pri-*miR164A* transcript level was low in the WT but high in *ein3* or *ORE1ox ein3* (Fig 1F). This result supports that EIN3 may also regulate *ORE1* by repressing the *miR164* function, as found previously [27]. We also found that the pri-*miR164A* transcript level was higher in *ORE1ox* line than that in WT (Fig 1F), suggesting a possible feedback upregulation of *miR164* by highly accumulated *ORE1*. Taken together, EIN3 positively regulates *ORE1* expression through 2 distinct ways during ethylene-mediated chl degradation: 1) directly binding to the *ORE1* promoter and activating its expression, and 2) indirectly inhibiting the expression of *miR164* and thereby activating *ORE1*.

### EIN3 regulates chl degradation by directly activating major *CCGs*

**Fig 2:**
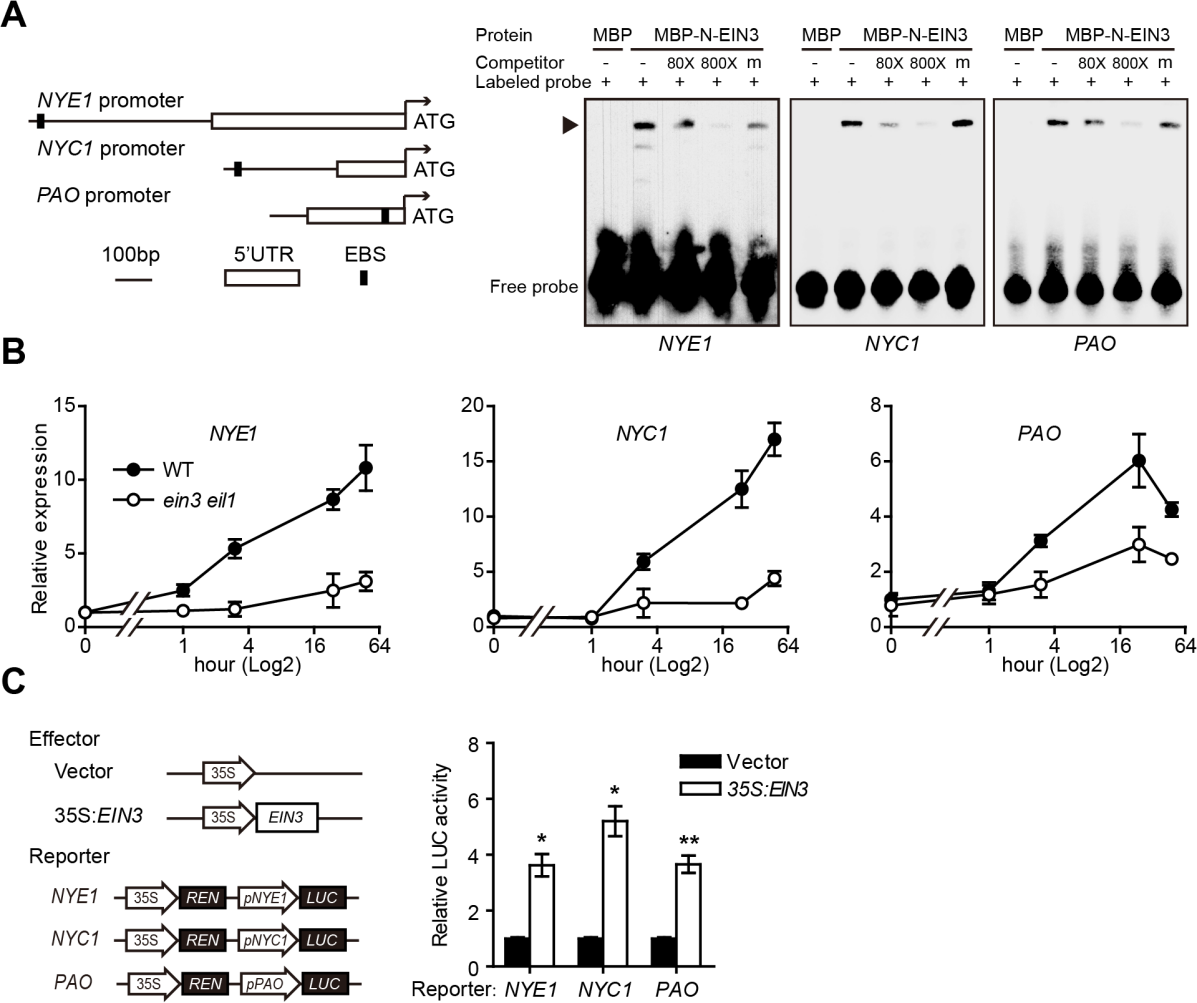
EIN3 directly associates with and transactivates the promoters of *NYE1*, *NYC1*, and *PAO*. (A) Left panel: Schematic diagrams of EIN3 binding site (EBS) in the promoter or 5’-UTR regions of *NYE1*, *NYC1*, and *PAO*. Right panel: EIN3 physically interacts with the promoters of *NYE1*, *NYC1*, and *PAO* in EMSA. About 30-bp DNA fragments containing the EBS in the promoters or 5’-UTR of *NYE1*, *NYC1*, and *PAO* were used as probes for EMSA. Mutated version of competitor DNA (m) was added in 800-fold molar excess. “‒” and “+” represent absence or presence, respectively. Triangle indicates the DNA-protein complex. (B) Kinetic analysis of *NYE1*, *NYC1*, and *PAO* expression in leaves of WT and *ein3 eil1* in response to ethylene. Experiments were performed as in Fig 1A. The expression of each corresponding gene in the WT at 0 hr was set to 1. Data are mean ± SEM of 3 biological replicates with technical duplicates for each. (C) Left panel: Schematic diagrams of effector and reporter constructs used in the transient dual-luciferase assays. CaMV 35S promoter driving *EIN3* (*35S*:*EIN3*) was used as effector, and the empty vector was used as a control. The dual-luciferase reporter constructs consist of *35S* driving *Renilla* luciferase (REN) reporter gene for internal normalization, and the promoters of *NYE1* (2012 bp), *NYC1* (493 bp), *PAO* (365 bp) driving firefly luciferase (LUC) reporter gene. Right panel: transient dual-luciferase assay of EIN3 transactivating the promoters of *NYE1*, *NYC1*, and *PAO* in *Arabidopsis* protoplasts. The procedure was as in Fig 1B. Data are mean ± SEM of at least 3 biological replicates. * *p* < 0.05, ** *p* < 0.01 (*t*-test).

The above result indicated that ORE1 is a direct target of EIN3 during ethylene-mediated leaf senescence. Previously Li et al. found that the leaves of *ein3 eil1* showed delayed senescence and a stay-green phenotype with ACC treatment [27]. Our results also confirmed the stay-green phenotypes in both the whole plant and detached leaves of *ein3 eil1* with ethylene treatment (S1 Fig). Thus, EIN3 and EIL1 may play an indispensable role in chl degradation during ethylene-mediated senescence. The process of chl degradation is regulated by a number of *CCGs*, and some may represent direct targets of EIN3. EIN3/EIL1 transcription factors were reported to bind to a consensus DNA sequence of A[CT]G[AT]A[CT]CT [34,35]. We performed an *in silico* analysis to specifically scan for the consensus sequence in the non-coding regions of those *CCGs* and identified some putative EBS in the promoter or 5’-UTR regions of *NYE1*, *NYC1* and *PAO* (Fig 2A). Therefore, we studied whether EIN3 could directly target these 3 *CCGs* to regulate their expression. EMSA results showed that the negative control MBP protein could not retard the mobility of biotin-labeled probes, whereas the MBP-N-EIN3 fusion protein caused a clear shift, which indicates the binding of the protein to probes (Fig 2A). Excessive amount of unlabeled probes successfully competed with the labeled probes in a dose-dependent manner, whereas excessive amount of mutated probes could not compete for binding (Fig 2A). Therefore, EIN3 physically bound to the promoters of *NYE1*, *NYC1* and *PAO in vitro*.

To examine the effect of EIN3/EIL1 on the expression of *NYE1*, *NYC1* and *PAO*, we analyzed the kinetic expression of these *CCGs* in response to ethylene treatment and found that the transcript levels of *NYE1*, *NYC1* and *PAO* were all greatly induced in WT plants; however, the ethylene induction of these *CCGs* was largely abolished in the *ein3 eil1* double mutant (Fig 2B and S2 Fig). These results strongly suggest that EIN3 and EIL1 may positively regulate the transcription of these *CCGs* in ethylene-mediated chl degradation. To further investigate whether EIN3 indeed transcriptionally activated *NYE1*, *NYC1* and *PAO*, transient dual-luciferase assays were carried out in *Arabidopsis* protoplasts. The *NYE1*, *NYC1* and *PAO* promoters, which are 2012, 493 and 365 bp in length, respectively, were individually fused with the firefly luciferase (LUC) gene [36] and served as reporter constructs. Each reporter construct contained a separate expression cassette (*35S*:*REN*) with the *Renilla* luciferase (REN) gene under the control of a CaMV 35S promoter and functioned as an internal control to normalize the expression of each reporter gene (Fig 2C). A construct with *35S* promoter driving the full-length *EIN3* cDNA (*35S*:*EIN3*) was used as an effector, and the empty vector was included as a control (Fig 2C). The reporter and effector constructs were co-transformed into *Arabidopsis* protoplasts prepared from WT plants and luciferase activity (LUC/REN) was detected. Overexpression of *EIN3* increased luciferase activity of each reporter as compared with the corresponding empty control (Fig 2C), which further demonstrates that EIN3 activated *NYE1*, *NYC1* and *PAO* at transcriptional levels. Thus, EIN3 directly bound to promoters of the *CCGs*, *NYE1*, *NYC1*, and *PAO* and activated their transcription, which indicates that EIN3 play a crucial regulatory role during ethylene-mediated chl degradation.

### ORE1 directly activates *CCGs*

**Fig 3:**
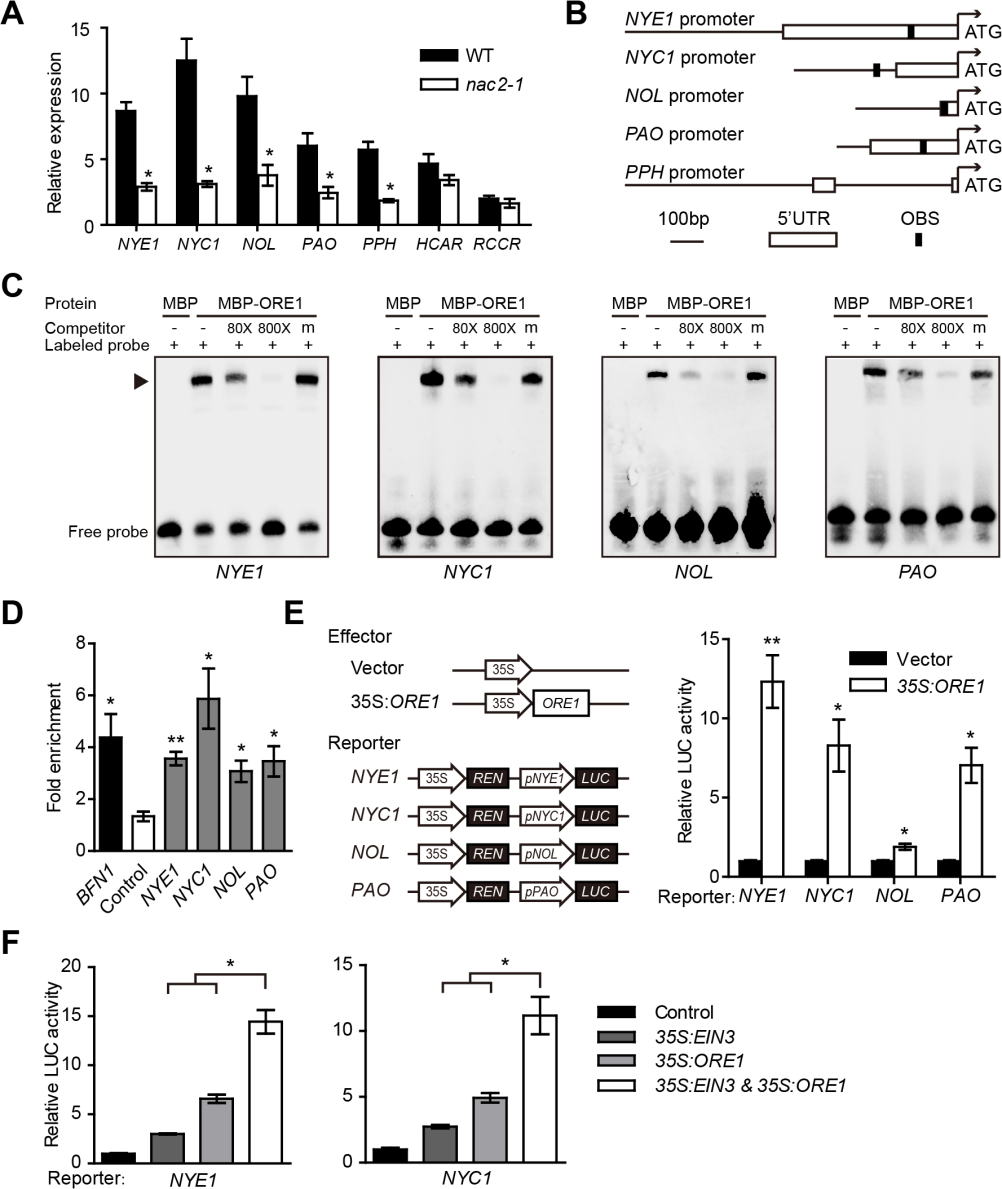
ORE1 directly activates the expression of *NYE1*, *NYC1*, *NOL* and *PAO*. (A) Relative expression of *CCG*s in leaves of WT and *ein3 eil1*with ethylene treatment for 24 hr. Gene expression was relative to that in the WT at 0 hr. Data are mean ± SEM of 3 biological replicates with technical duplicates for each. * *p* < 0.05 (*t*-test). (B) Schematic diagrams of ORE1 binding site (OBS) in the promoter or 5’-UTR regions of *NYE1*, *NYC1*, *NOL* and *PAO*. (C) ORE1 physically interacts with the promoters of *NYE1*, *NYC1*, *NOL*, and *PAO* in EMSA. About 30-bp DNA fragments containing the OBS in the promoter or 5’-UTR regions of *NYE1*, *NYC1*, *NOL*, and *PAO* were used as probes for EMSA, with purified MBP or MBP-ORE1 protein expressed in *E*. *coli*. “‒” and “+” represent in absence or presence, respectively. “m” represents mutated competitor. Triangle indicates the DNA-protein complex. (D) ORE1 associated with the promoters of *NYE1*, *NYC1*, *NOL*, and *PAO* in ChIP-qPCR assay. Chromatins isolated from *35S*:*ORE1-GFP* transgenic line and WT control were immunoprecipitated with anti-GFP antibody followed by qPCR to amplify regions covering the putative ORE1 binding sites. Input sample was used to normalize the qPCR results in each ChIP sample. *BFN1*, reported as a direct target of ORE1, was used as a positive control. A retrotransposon (At4g03770) located within the heterochromatic region associated with di-methylated H3-K9 was used as a negative control. Fold enrichment was presented as a ratio of normalized results from *35S*:*ORE1-GFP* plants and WT. Data are mean ± SEM of at least 3 technical replicates. * *p* < 0.05, ** *p* < 0.01 (*t*-test). The experiment was repeated twice with similar results. (E) Left panel: Schematic diagrams of effector and reporter constructs used in the transient dual-luciferase assays. CaMV 35S promoter driving *ORE1* (*35S*:*ORE1*) was used as effector, and empty vector as a negative control. A 309-bp fragment upstream from ATG of *NOL* was used to make the *pNOL*:*LUC* reporter construct and all other reporters were as in Fig 2C. Right panel: Transient dual-luciferase assay of ORE1 transactivates the promoters of *NYE1*, *NYC1*, *NOL*, and *PAO* in *Arabidopsis* protoplasts. The procedure was as in Fig 2C. Data are mean ± SEM of at least 3 biological replicates. * *p* < 0.05, ** *p* < 0.01 (*t*-test). (F) EIN3 and ORE1 transactivate the promoters of *NYE1* and *NYC1* in *Arabidopsis* protoplasts in an additive manner. The transient expression procedure, and the constructs used for the assay were as in Figs 2C and 3E. The amount of each effector was half that used in Figs 2C and 3E. The same amount of corresponding empty vector was used if one effector was absent in a transformation so that the total amount of plasmids was the same among all assays. Data are mean ± SEM of at least 3 biological replicates. * *p* < 0.05 (*t*-test).

*ORE1/NAC2* is a direct target of EIN3 in both age-dependent [31] and ethylene-mediated leaf senescence. The *ORE1* mRNA level was rapidly induced in the WT but not the *ein3 eil1* mutant with ethylene treatment (Fig 1A). Moreover, both the attached and detached leaves of *nac2-1*, a T-DNA insertion mutant of *ORE1*, showed a stay-green phenotype under ethylene treatment (S3 Fig), so ORE1 may also play a key role in ethylene-mediated chl degradation through CCGs. We checked the transcript levels of all *CCGs* in the *nac2-1* mutant and found significantly repressed transcript levels of *NYE1*, *NYC1*, *NOL*, *PAO* and *PPH* with ethylene treatment (Fig 3A), so ORE1 is required for full induction of these *CCGs*. As a transcription factor, ORE1 was reported to bind to consensus DNA sequences of [ACG][CA]GT[AG]N{5,6}[CT]AC[AG] [29] or T[TAG][GA]CGT[GA][TCA][TAG] [37]. Among the promoters or 5’-UTR regions of *NYE1*, *NYC1*, *NOL*, *PAO* and *PPH*, all except *PPH* contain either of these two consensus putative ORE1 binding sites (OBS) (Fig 3B). Therefore, ORE1 may directly target these *CCGs* to regulate their expression. To test this possibility, we used EMSA with recombinant MBP-ORE1 proteins and DNA fragments of each promoter covering the putative OBS that were close to the ATG of each gene and found specific binding of MBP-ORE1 to the promoters of *NYE1*, *NYC1*, *NOL* and *PAO* (Fig 3C). As well, ChIP-qPCR assay revealed the association of ORE1 protein with these four promoters in *35S*:*ORE1-GFP* transgenic *Arabidopsis* (Fig 3D).

The direct transactivation effect of ORE1 on the expression of *CCGs* was further confirmed by transient dual luciferase assay in *Arabidopsis* protoplasts, with a construct overexpressing *ORE1* (*35S*:*ORE1*) as an effector and promoters of *NYE1*, *NYC1*, *NOL* and *PAO* driving *LUC* as reporters (Fig 3E). It showed that ORE1 significantly transactivated the promoters of *NYE1*, *NYC1*, *NOL* and *PAO* (Fig 3E). Interestingly, *EIN3* and *ORE1* had additive effects in activating the promoters of both *NYE1* and *NYC1* (Fig 3F). Therefore, both EIN3 and ORE1 may actively regulate the transcription of *NYE1* and *NYC1* by directly binding to their promoters and contributing to chl degradation.

### The EIN3- and ORE1-mediated chl degradation during ethylene-induced senescence is NYE1-dependent

**Fig 4:**
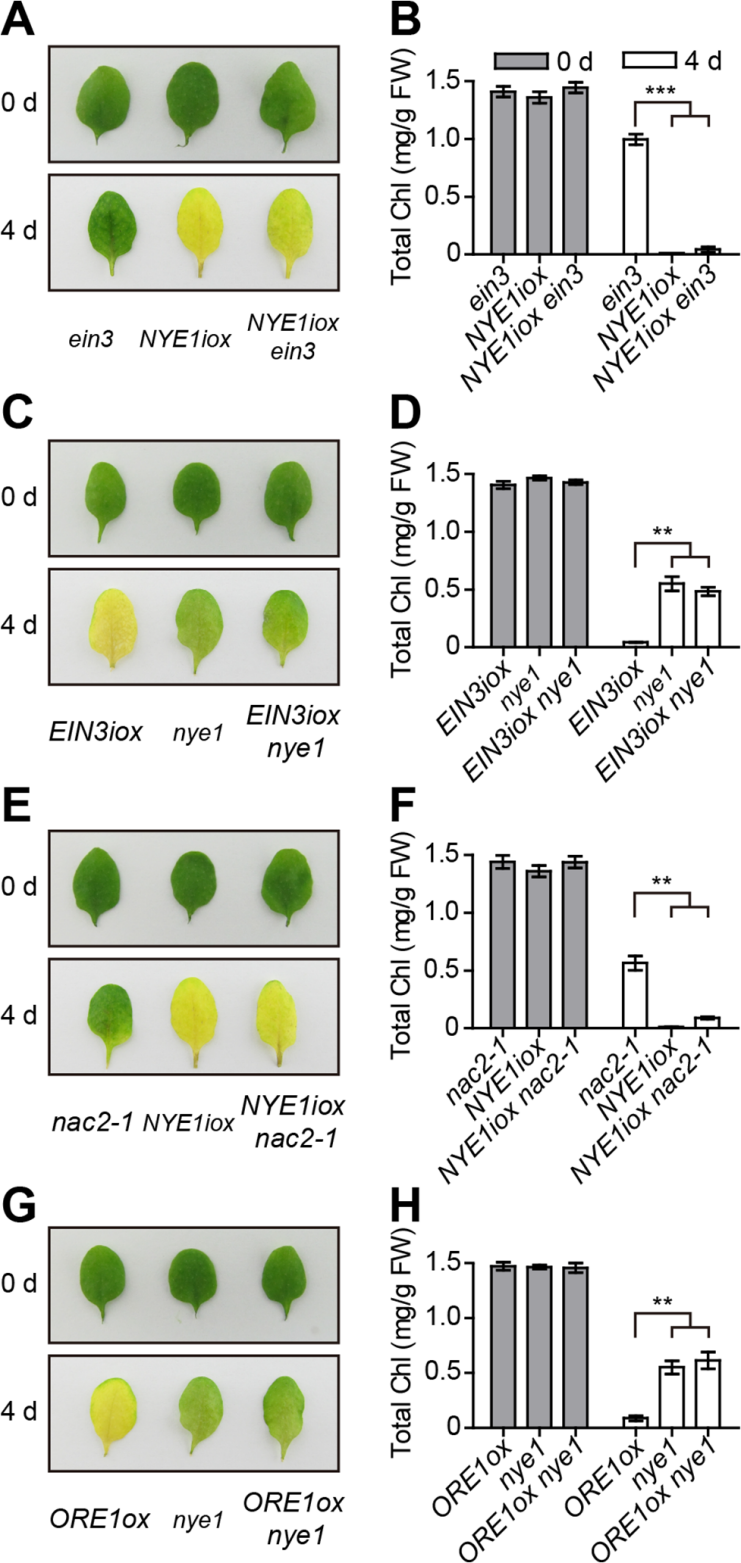
Ethylene-induced chl degradation through *EIN3* and *ORE1* is *NYE1*-dependent. (A) Inducible overexpression of *NYE1* reversed the stay-green phenotype of e*in3* mutant under ethylene treatment. The dexamethasone (DEX)-inducible *NYE1* line (*NYE1iox*) was crossed with *ein3* to obtain *NYE1iox ein3*. The leaves of each indicated genotype were sprayed with 15 μmol/L DEX to induce *NYE1* expression and underwent ethylene treatment for 4 d. (B) Quantification of total chl content in leaves of each indicated genotype and time point shown in (A). (C) The loss-of-function *nye1* mutant repressed chl degradation of the inducible overexpression of *EIN3* transgenic line during ethylene-induced senescence. The estradiol (EST)-inducible *EIN3* line *EIN3iox* was crossed with *nye1* to obtain *EIN3iox nye1*. Leaves were sprayed with 100 μmol/L EST to induce *EIN3* expression and underwent ethylene treatments for 4 d. (D) Quantification of total chl content in leaves of each indicated genotype and time point shown in (C). (E) Inducible overexpression of *NYE1* reversed the stay-green phenotype of *nac2-1* mutant under ethylene treatment. The DEX-inducible *NYE1* line (*NYE1iox*) was crossed with *nac2-1* mutant to obtain *NYE1iox nac2-1*. The treatments were as in (A) except for different plant genotypes. (F) Quantitative analysis of total chl content in leaves of each indicated genotype and time point shown in (E). (G) The loss-of-function *nye1* mutant repressed chl degradation caused by overexpression of *ORE1* during ethylene-induced senescence. The leaves underwent ethylene treatment for 4 d. (H) Quantitative analysis of total chl content in leaves of each indicated genotype and time point shown in (G). For (A), (C), (E) and (G), the photographs were taken before (0 d) or after (4 d) induction and treatment. For (B), (D), (F) and (H), data are mean ± SEM (n > 4). ** *p* < 0.01, *** *p* < 0.001 (*t*-test). The total chl contents of all tested lines were similar before induction and treatment.

The above data showed that both EIN3 and ORE1 directly promoted the transcription of three major *CCGs*, *NYE1*, *NYC1*, and *PAO*, during ethylene-mediated chl degradation. We then analyzed the relationship between CCGs and EIN3 or ORE1. Since NYE1 was reported to be essential for recruiting major CCEs into a possible multi-protein complex in senescing chloroplasts [11], and *NYE1* responded earlier than did *NYC1* and *PAO* to ethylene treatment and the responsiveness was significantly repressed in *ein3 eil1* and *nac2-1* mutants (Fig 2B and S4 Fig), and *nye1* mutant showed stronger stay-green phenotype than that of *nyc1* and *pao* mutants (Fig 4C and S7 Fig), NYE1 was then selected as a representative of CCGs. To investigate the relationship among NYE1, EIN3 and ORE1 in regulating ethylene-mediated chl degradation, we generated plant lines with different combination of these genotypes and measured their chl contents before (0 d) and after (4 d) ethylene treatment. The total chl contents of all tested lines were similar before any treatment, which were consistent with their leaf colors (Fig 4). An inducible *NYE1* overexpression transgenic line (*NYE1iox*), which displayed an early-yellowing phenotype with ethylene treatment (Fig 4A and S5 Fig), was crossed with *ein3* mutant. *ein3* exhibited a stay-green phenotype with high chl content upon ethylene treatment (Fig 4A and 4B). Inducible expression of *NYE1* efficiently reversed the stay-green phenotype of *ein3* (Fig 4A and 4B). We also crossed an inducible *EIN3* line (*EIN3iox*) to *nye1* mutant. *EIN3iox* line displayed an early-yellowing phenotype with low chl content upon ethylene treatment (Fig 4C and 4D), which was opposite to that of *nye1* mutant, whereas mutation of *NYE1* suppressed EIN3-induced early-yellowing phenotype (Fig 4C and 4D). Similarly, inducible expression of *NYE1* reversed the stay-green phenotype caused by *ORE1/NAC2* mutation (Fig 4E and 4F), and *NYE1* mutation repressed the early-yellowing phenotype caused by overexpression of *ORE1* (Fig 4G and 4H). These results suggest that the ethylene-induced chl degradation through *EIN3* and *ORE1* depends on functional *NYE1* gene.

### ORE1 directly activates the expression of *ACS2*

**Fig 5:**
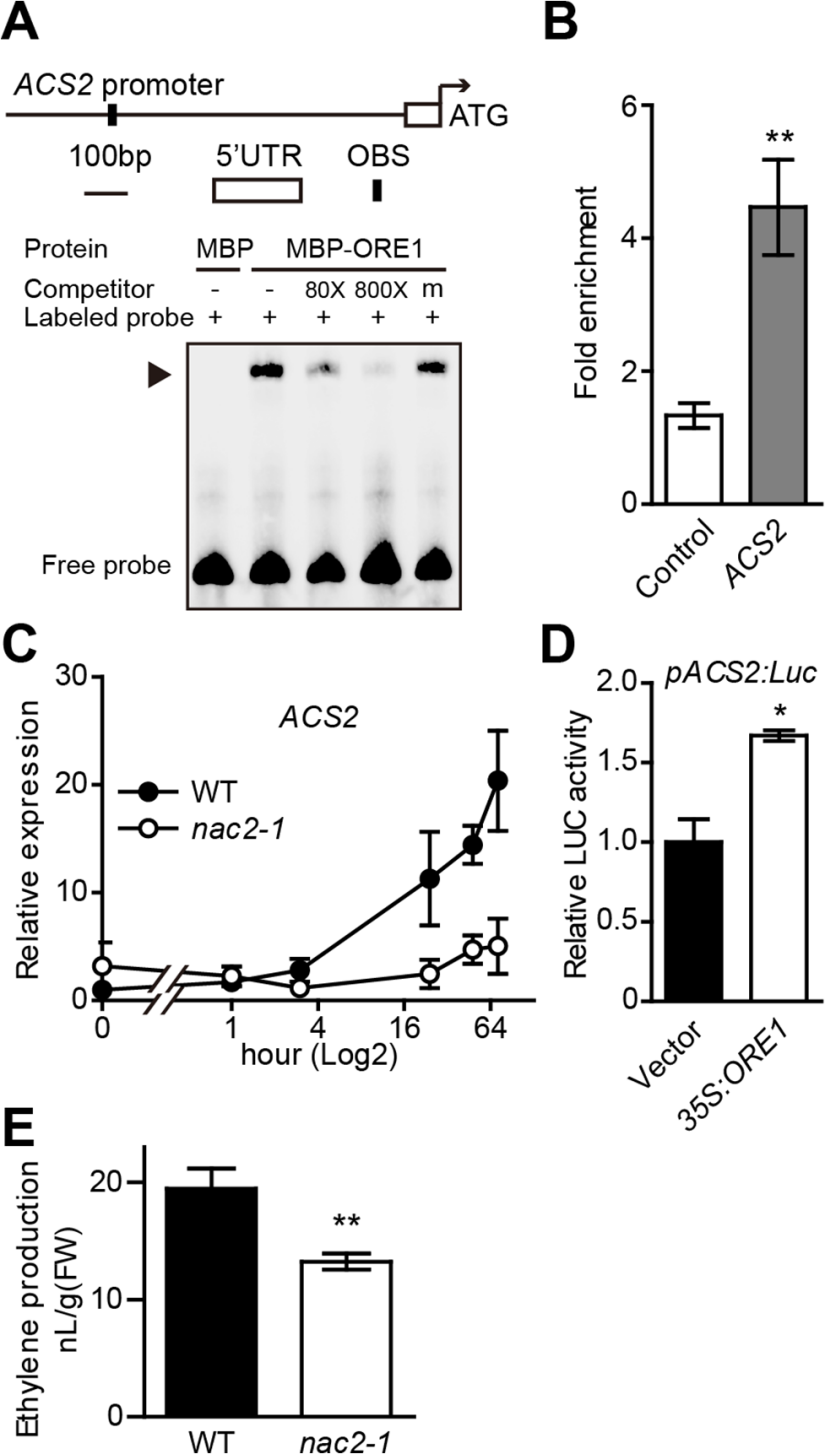
ORE1 is associated with *ACS2* promoter and transcriptionally activates its expression. (A) EMSA detection of binding of ORE1 to *ACS2* promoter *in vitro*. A 45-bp *ACS2* promoter fragment containing the putative ORE1 binding site was biotin-labeled and used as a probe. Purified MBP-ORE1 protein expressed in *E*. *coli* was used in EMSA. MBP was included as a negative control. “‒” and “+” represent absence or presence, respectively. “m” represents mutated competitor. Triangle indicates the DNA-protein complex. (B) ChIP-qPCR analysis of ORE1 binding to *ACS2* promoter *in vivo*. The *ACS2* promoter region containing a putative ORE1 binding site was amplified to detect the enrichment. The ChIP procedure and qPCR data processing were as described in Fig 3D. Data are mean ± SEM of at least 3 technical replicates. ** *p* < 0.01 (*t*-test). The experiment was repeated twice with similar results. (C) Kinetic analysis of *ACS2* expression in WT and *nac2-1* with ethylene treatment for various periods of time (0, 1, 3, 24, 48, 72 hr). Expression in WT at 0 hr was set to 1. Data are mean ± SEM of 3 biological replicates with technical duplicates for each. (D) Transient dual-luciferase assay of transactivation of the *ACS2* promoter by ORE1 in *Arabidopsis* protoplasts. A 1019-bp *ACS2* promoter fragment covering the putative ORE1 binding site was used for making the *pACS2*:*LUC* reporter construct. The *35S*:*ORE1* effector construct was described in Fig 3E. Data are mean ± SEM of 3 biological replicates. * *p* < 0.05 (*t*-test). (E) Ethylene production in the leaves of WT and *nac2-1* during senescence. Data are mean ± SEM (n = 9). ** *p* < 0.01 (*t*-test). The experiment was repeated twice with similar results.

Previous data suggested a positive role of ORE1 in ethylene-mediated chl degradation. To identify possible ORE1 direct targets by scanning for putative ORE1 binding sites in the promoter regions of candidate genes induced by senescence, we identified some *CCGs* as putative direct targets but also located a putative ORE1 binding site [ACG][CA]GT[AG]N{5,6}[CT]AC[AG] [29] in the promoter region of *ACS2*, an ACC synthesis gene with the mRNA level increased with leaf aging (Fig 5A). We then examined the interaction of ORE1 protein with the promoter of *ACS2*. ChIP assay, with the *35S*:*ORE1-GFP* transgenic line in *Arabidopsis*, showed an approximately four-fold enrichment of the *ACS2* promoter region harboring the putative ORE1 binding site (Fig 5B). The direct binding of ORE1 protein to the *ACS2* promoter was further confirmed by EMSA (Fig 5A). To examine the effect of ORE1 on the expression of *ACS2* during ethylene-induced senescence, we analyzed the kinetic expression of *ACS2* with ethylene treatment. The transcript level of *ACS2* was greatly induced in the leaves of WT plants at a later stage of the treatment (Fig 5C); however, the ethylene induction of *ACS2* was repressed in the loss-of-function *nac2-1* mutant (Fig 5C), indicating that ORE1 may positively regulate the expression of *ACS2*. This finding was further confirmed by a dual luciferase assay in *Arabidopsis* protoplasts, with *ACS2* promoter activity being monitored with a *pACS2*:*LUC* reporter construct. Overexpression of *ORE1* significantly increased the activity of *ACS2* promoter (Fig 5D). To further determine whether *ORE1* mutation affects ethylene biosynthesis during senescence, we quantified ethylene production in the leaves of both WT and *nac2-1* and found that *nac2-1* leaves produced about 30% less ethylene within a period of 72 hr after detachment, in comparison to that produced by WT leaves (Fig 5E). These results support that ORE1 promotes ethylene synthesis via directly regulating the expression of *ACS2* likely in a positive feedback manner.

## Discussion

Our data reveal novel functions of existing transcription factors, EIN3 and ORE1, involved in activating the expression of several *CCGs*, which in turn accelerate ethylene-mediated chl degradation in *Arabidopsis*. We showed that EIN3 promotes ethylene-mediated chl degradation by activating the expression of some key *CCGs*, namely *NYE1*, *NYC1* and *PAO*, via directly binding to their promoters. NYE1 was reported to be essential for recruiting CCEs into a possible multi-protein complex in the beginning of chl breakdown in senescing chloroplasts [11]. NYC1 is a chl *b* reductase that catalyzes the first step of chl degradation [6]. PAO is responsible for the ring opening reaction of the porphyrin macrocycle and is the key enzyme of the chl degradation pathway [10]. We found that EIN3 proteins bound to specific regions of the *NYE1*, *NYC1* and *PAO* promoters (Fig 2A) and the relative expression of these genes was induced by ethylene in WT plants, but the expression considerably reduced in the *ein3 eil1* double mutant even with ethylene treatment (Fig 2B). In addition, overexpression of EIN3 transactivated the expression of *NYE1*, *NYC1* and *PAO* promoters (Fig 2C). Above all, we identified novel EIN3 direct targets, NYE1, NYC1 and PAO, which are the major CCGs in chl degradation pathway.

EIN3 was previously shown to promote leaf senescence by accumulating mRNA of *ORE1* which has been reported to play a central role in leaf senescence and cell death [27–29,31,38]. We further found that ethylene-induced *CCG* expression was repressed in the loss-of-function *nac2-1* mutant (Fig 3A and S4 Fig), so ORE1/NAC2 may play a positive role during chl degradation. Transient dual luciferase assay further confirmed that overexpression of ORE1 significantly transactivated the promoter activity of *NYE1*, *NYC1*, *NOL* and *PAO* (Fig 3E). Moreover, *in vivo* ChIP-qPCR and *in vitro* EMSA suggested that ORE1 directly bound to the promoters of these *CCGs* and activated their transcription (Fig 3C and 3D). Altogether, these results reveal four *CCGs* are novel direct targets of ORE1 in chl degradation during leaf senescence. We further found that EIN3 and ORE1 shared *NYE1*, *NYC1* and *PAO* as common targets, and ORE1 targeted an additional *CCG*, *NOL*. In addition, EIN3 and ORE1 promoted the expression of *NYE1* and *NYC1* in an additive manner (Fig 3F). These results suggest that ORE1, a directly target of EIN3, strengthen and broaden the signal from EIN3.

**Fig 6:**
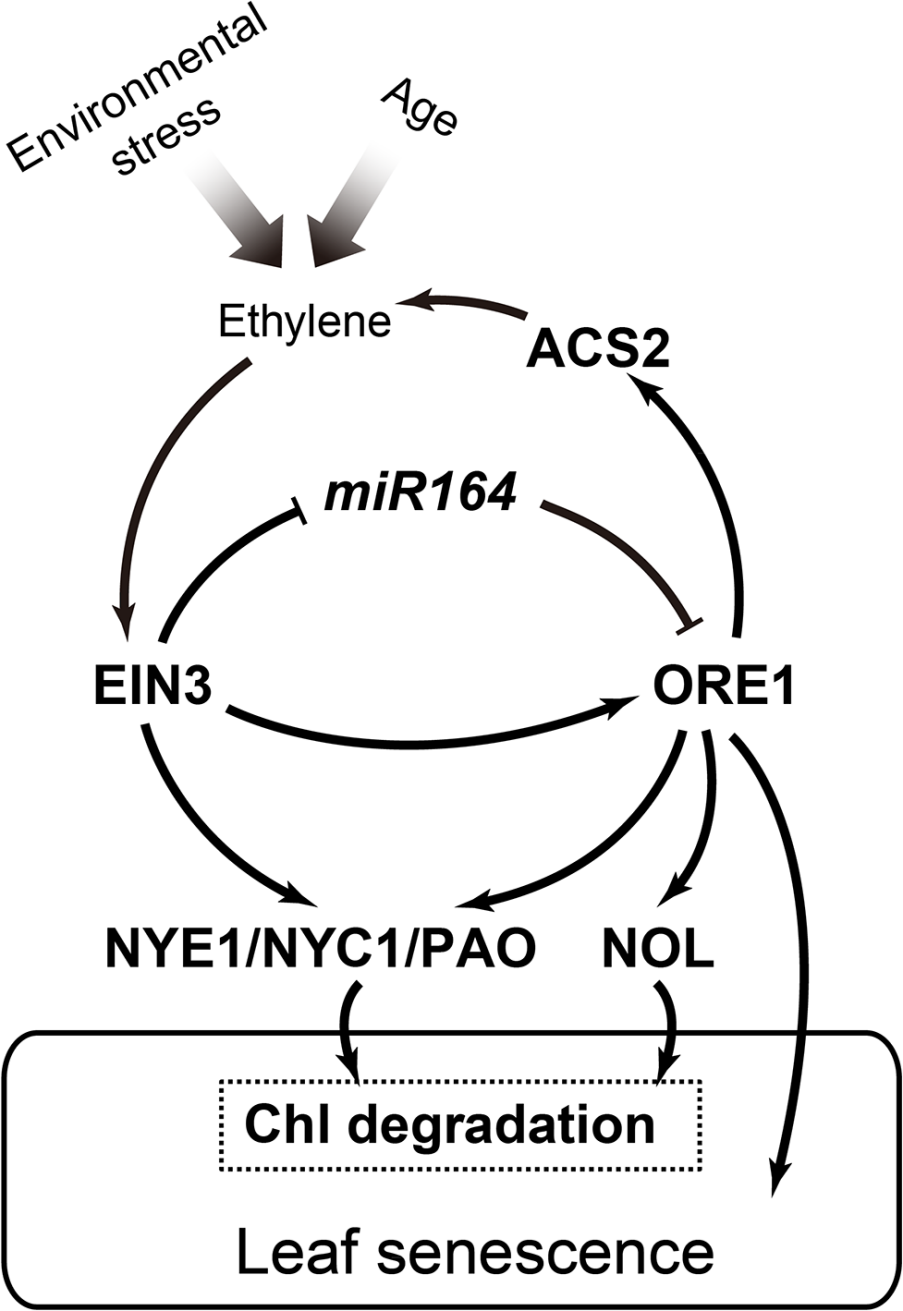
A working model of the EIN3-ORE1-CCGs coherent feed-forward loop in regulation of ethylene-mediated chl degradation. According to our study and previous reports [27,31], we propose a coherent feed-forward loop that involves EIN3 and ORE1 in regulating ethylene-mediated chl degradation. EIN3 directly represses the transcription of *miR164*, which negatively regulates *ORE1* at the post-transcriptional level. Meanwhile, EIN3 can directly bind to the *ORE1* promoter and induce *ORE1* transcription. Three *CCGs*, *NYE1*, *NYC1*, and *PAO*, are the direct targets of EIN3. As a transcription factor downstream of EIN3, ORE1 shares these 3 common direct targets with EIN3. However, ORE1 also has its own distinct target, *NOL*, during the regulation of chl degradation. The broad range of expression of *CCGs* leads to chl degradation, the early step of leaf senescence. In addition, ORE1 directly activates the expression of *ACS2*, which presumably triggers a positive feedback regulation of ethylene synthesis. Arrows and bars represent positive and negative regulations, respectively.

Our data support that EIN3 promoted ethylene-mediated chl degradation by (1) directly activating the expression of *CCGs* or (2) indirectly activating the intermediate regulator ORE1, which in turn activates more *CCGs* and enhances their expressions. EIN3, ORE1, and CCGs constitute a coherent feed-forward loop in the regulation of ethylene-mediated chl degradation during leaf senescence (Fig 6). We noticed that ethylene-induced *NYE1* mRNA accumulation increased earlier than did *NYC1* and *PAO* levels (Fig 2B), indicating that different *CCGs* require different threshold values of EIN3 and/or ORE1 to initiate their transcription. Considering that NYE1 was reported to recruit CCEs such as NYC1 and PAO during chl degradation, the earlier induction of *NYE1* by EIN3 and/or ORE1 might be necessary for its function. This fine-tuning mechanism may allow plants to robustly respond to ethylene-mediated chl degradation.

In addition to EIN3 and ORE1 being able to bind to the promoters of *NYE1*, *NYC1* and *PAO* to modulate ethylene-mediated chl catabolism, ABSCISIC ACID INSENSITIVE3 (ABI3), a B3 domain transcription factor that confers desiccation tolerance during seed maturation, regulates *NYE1* expression during seed degreening [39]. ABI5 and ENHANCED EM LEVEL (EEL), two Group A bZIP transcription factors in the ABA signaling pathway, can bind to the promoters of *NYE1* and *NYC1* to directly accelerate chl degradation [4]. A more recent paper showed that the Phytochrome-interacting factor4 (PIF4), a bHLH transcriptional factors of light signal transduction pathway, binds to the promoter of *NYE1* to activate chl catabolism [40]. Therefore, EIN3 and ORE1, together with other type of transcription factors from other signaling pathways, may promote key chl catabolic genes, such as *NYE1*, during chl breakdown. This mechanism allows plants to effectively and coordinately respond to environmental changes or stress conditions by hormone-mediated chl degradation.

We detected a lower level of *ACS2* transcript level in the loss-of-function *nac2-1* mutant than that in WT plants (Fig 5C) but a higher level in the *ORE1* overexpression line (S6 Fig), suggesting that ORE1 might be a positive regulator of *ACS2* expression. Transient dual luciferase assay indeed revealed that overexpression of ORE1 transactivated the expression of *ACS*2 (Fig 5D). Furthermore, a putative ORE1 binding site was found in the promoter of *ACS2*, and EMSA and ChIP-qPCR results suggested that ORE1 directly bound to the putative ORE1 binding site in the *ACS2* promoter (Fig 5A and 5B). Moreover, we quantified ethylene production in detached leaves of WT and the *nac2-1* mutant plants and found that *nac2-1* indeed produced less ethylene than did WT (Fig 5E). Our data revealed that in addition to directly accelerating chl degradation and leaf senescence, ORE1 promotes ethylene synthesis by activating the expression of *ACS2*. In turn, ethylene further accelerates chl degradation and leaf senescence (Fig 6). The complicated network of interactions involving both positive feed-forward loop (EIN3-ORE1-CCGs) and positive feed-back regulation of ethylene synthesis (ORE1-ACS2) would likely lead to an irreversible process. This may facilitate plants to activate all available approaches to quickly trigger senescence processes during their final developmental stages.

In conclusion, our results reveal that EIN3 protein promotes expression of the *CCGs*, *NYE1*, *NYC1* and *PAO* by directly binding to their promoters to advance ethylene-mediated chl degradation. Meanwhile, one of the EIN3 target genes, *ORE1/NAC2* [31], can also directly activate the expression of *NYE1*, *NYC1* and *PAO* as well as other *CCGs* such as *NOL*. EIN3 and ORE1 additively activate the expression of *NYE1* and *NYC1*. In addition, ORE1 activates the expression of *ACS2*, presumably triggering a positive feedback regulation of ethylene production. Collectively, our work reveals a coherent feed-forward loop, involving EIN3, ORE1 and CCGs, which efficiently regulates ethylene-mediated chl degradation during leaf senescence in *Arabidopsis*.

## Materials and Methods

### Plant materials and growth conditions

All plants, including the WT, mutants and transgenic lines, were in *Arabidopsis thaliana* ecotype Columbia-0 (Col-0) background. The mutants *ein3 eil1* [41], *nye1* [12] and the *EIN3iox* transgenic line [42] were described previously. To generate *NYE1iox* transgenic line, the full-length *NYE1* coding sequence (CDS) was PCR amplified, using WT cDNA as template, and cloned into the vector pTA7002 [43]. The final construct was transformed into *nye1* mutant using the floral-dipping method [44]. The *ORE1ox* line [45] and *35S*:*ORE1-GFP* line [29] were kindly provided by Feng Ming (Fudan University, China) and Bernd Mueller-Roeber (University of Potsdam, Germany), respectively. The mutant *nac2-1* (SALK_090154) was obtained from the ABRC and the homozygosity of this line was confirmed by PCR-based genotyping. *NYE1iox ein3*, *EIN3iox nye1*, *NYE1iox nac2-1* and *ORE1ox nye1* were generated by genetic crossing. Homozygous lines were genotyped by PCR. Primers for genotyping are listed in S1 Table.

After surface sterilization, imbibed seeds were stratified for 3 days at 4°C to synchronize germination. Plants were grown in an environmentally controlled chamber at 22°C-24°C, at light intensity of approximately 120 μmol m^-2^ s^-1^ under a 16-hr light/8-hr dark photoperiod.

### Ethylene-induced senescence

The third and fourth rosette leaves from 4-week-old plants were used for ethylene-induced senescence assay. Leaves were detached and placed on two layers of moist filter papers in Petri dishes to maintain moisture and were placed in a sealed glass desiccator. Ethylene was released by adding 1M ethephon stock solution into 5 mM Na_2_HPO_3_ buffer at the bottom of the desiccator [46]. The final concentration of ethylene was 100 μL/L. For kinetic analysis of gene expression, Petri dishes were placed in a sealed container that was injected with ethylene gas at a final concentration of 100 μL/L. Ethylene treatment was performed at 22°C to 24°C under a normal 16-hr/8-hr photoperiod for 0, 1, 3, 24, and 48 hr, unless otherwise stated.

### Chl content measurement

Chl content was measured as previously described [47]. Briefly, leaves were incubated in DMSO at 65°C for 30 minutes, and absorbance was measured at 663 and 645nm. The concentration of total chl was calculated as follows: total chl (mg/L) = 20.2×D_645_ + 8.02×D_663_. The chl content was converted to microgram per gram fresh weight of leaf tissue (mg/g FW).

### qRT-PCR

Total RNA was extracted by use of TRIzol reagent (Invitrogen) according to the manufacturer's instructions. First-strand cDNA was synthesized with the PrimeScript RT Master Mix (TaKaRa, China) and then used as templates for quantitative RT-PCR (qRT-PCR) with SYBR Premix Ex Taq II (Perfect Real-Time; TaKaRa, China) and the MyiQ2 Real Time PCR Detection System (Bio-Rad, Hercules, CA). *ACT2* was an internal control for normalization. Primers used for qRT-PCR are listed in S1 Table.

### Protein expression and electrophoretic mobility shift assay (EMSA)

For protein expression and purification, 1 to 942 bp of *EIN3* CDS containing the DNA-binding domain and full-length CDS of *ORE1/ANAC092* were cloned into pMAL-c5g (New England Biolabs). The empty vector pMAL-c5g was used for MBP expression alone as a negative control in EMSA. Plasmids were transformed into *Escherichia coli* strain Rosetta (DE3) pLysS (Merck). The expression of proteins was induced by 1mM isopropyl thio-β-D-galactoside (IPTG) at 20°C for 10 hr in 200 mL LB medium. Cells were then collected and sonicated. Protein was purified with Amylose resin (New England Biolabs) following the manufacturer’s instructions.

EMSA was carried out using the LightShift Chemiluminescent EMSA Kit (Thermo Scientific). Briefly, 400 ng purified protein was incubated with 12.5 fmol 5’-biotin-labeled probe DNA and 1 μg poly (dI-dC) in binding buffer for 15 min. Binding reactions were resolved on a polyacrylamide gel and electrophoretically transferred to nylon membrane. The transferred DNA was cross-linked to membrane by use of CL-1000 Ultraviolet Crosslinker (UVP, Upland, CA, USA). Biotin-labeled DNA was detected by chemiluminescence and exposed with a ChemiScope 3500 Mini Imaging System (Clinx Science Instruments, China).

### Dual-luciferase transient expression assay in *Arabidopsis* protoplasts

To generate luciferase reporter constructs, the promoters of *ORE1* (1694 bp), *NYE1* (2012 bp), *NYC1* (493 bp), *NOL* (309 bp), *PAO* (365 bp), and *ACS2* (1019 bp) were amplified from Col-0 genomic DNA and cloned into the transient expression vector pGreenII 0800-Luc, with the expression of two cassettes—the target promoters driving a firefly luciferase (LUC) reporter gene and a CaMV 35S promoter driving an *Renilla* luciferase (REN) gene as an internal control [36]. To generate *35S*:*EIN3* effector construct, the full-length *EIN3* coding sequence (CDS) was PCR amplified, using WT cDNA as template, and cloned into pDONR221 (Invitrogen, Carlsbad, CA), then into the destination vector pEarleyGate203 [48] to get pEarley203-EIN3 by recombination. The *35S*:*ORE1* effector construct pHB-ANAC092 was described previously [45]. Empty vectors pEARLY203 and pHBwere controls, respectively. Primers for all constructs are listed in S1 Table.

*Arabidopsis* mesophyll cell protoplast isolation and transformation were as described [49]. Rosette leaves of 4-week-old plants were cut into leaf strips, then digested in an enzyme solution containing 1.5% (w/v) cellulase R10 and 0.4% (w/v) macerozyme R10 (Yakult Honsha, Tokyo). Plasmids were introduced into protoplasts by PEG-mediated transformation. Transformed protoplasts were incubated overnight, and firefly and *Renilla* luciferase activity was quantified using Dual-Luciferase Reporter Assay System (Promega, USA) and detected with a Synergy 2 multi-mode microplate (Bio-Tek) according to the manufacturer’s instructions.

### ChIP-qPCR

The third and fourth leaves of 5-week-old WT and *35S*:*ORE1-GFP* plants were harvested and cross-linked with 1% formaldehyde. ChIP was carried out using the EpiQuik Plant ChIP Kit (Epigentek, Brooklyn, NY, USA) with the antibody against GFP (ab290; Abcam). Input samples and immunoprecipitated samples were analyzed by qPCR. Primers flanking the ORE1 binding sites in *NYE1*, *NYC1*, *NOL*, *PAO* and *ACS2* promoters were used to detect ORE1 enrichment. Primers amplifying a fragment in the heterochromatic region (At4g03770) were used for a negative control. The primer sequences are listed in S1 Table. Anti-dimethyl H3-K9 antibody supplied by the ChIP kit was used as a negative experimental control, and no enrichment was detected in either WT or *35S*:*ORE1-GFP* plants among all tested regions compared with using IgG (no antibody was conjugated). ChIP-qPCR results were first normalized with input sample as follows: cycle threshold (Ct) = Ct_ChIP_ − Ct_Input_. Relative enrichment was then calculated by the ratio of normalized results from *35S*:*ORE1-GFP* plants and WT control.

### Quantification of ethylene production

The third and fourth rosette leaves of 4-week-old plants of WT and *nac2-1* were used for quantification of ethylene production. The detached leaves were weighed and incubated in 2 mL Agilent vial (clear glass) at 22°C to 24°C under a normal 16-hr/8-hr photoperiod. Ethylene produced within 72 hr period after detachment was measured with a sensitive laser-based ethylene detector (ETD-300, Sensor Sense BV, Nijmegen, the Netherlands) following the manufacturer’s instructions.

### Statistical analysis

Data are given as mean ± SEM and were analyzed by two-tailed Student’s *t*-test or one-way ANOVA. *p* < 0.05 was considered statistically significant.

### Accession numbers

Genes and their associated accession numbers in the *Arabidopsis* Genome Initiative or GenBank/EMBL are as follows: *EIN3* (AT3G20770), *EIL1* (AT2G27050), *ORE1*/*NAC2*/*ANAC092* (AT5G39610), *NAP* (AT1G69490), *miR164A* (AT2G47585), *NYE1/SGR* (AT4G22920), *NYC1* (AT4G13250), *NOL* (AT5G04900), *PAO* (AT3G44880), *PPH* (AT5G13800), *HCAR* (AT1G04620), *RCCR* (AT4G37000), *ACS2* (AT1G01480), *ACT2* (AT3G18780).

## Supporting Information

S1 Fig The stay-green phenotype of *ein3 eil1* mutant with ethylene treatment. (A) Whole plants of 4-week-old *ein3 eil1* mutant showed a stay-green phenotype compared to the wild type (WT) with 100 μL/L ethylene treatment for 4 d. (B) Detached third and fourth rosette leaves from 4-week-old WT and *ein3 eil1* plants treated with 100 μL/L ethylene for 4 d. (C) Quantitative analysis of total chl content in leaves of each genotype shown in (B). Data are mean ± SEM (n>4). *** *p* < 0.001 (*t*-test).
(TIF)

S2 Fig Kinetic expression of *NYE1*, *NYC1*, and *PAO* in the leaves of WT and *ein3 eil1* with mock (air) treatment. The expression level of each corresponding gene in the WT at 0 hr was set to 1. The scales of y-axes are consistent with that in Fig 2B. Data are mean ± SEM of 3 biological replicates.
(TIF)

S3 Fig The stay-green phenotype of *nac2-1* mutant with ethylene treatment. (A) Whole plants of 4-week-old *nac2-1* mutant showed a stay-green phenotype compared to the WT with 100 μL/L ethylene treatment for 4 d. (B) Detached third and fourth rosette leaves from 4-week-old WT and *nac2-1* plants treated with 100 μL/L ethylene for 4 d. (C) Quantitative analysis of total chl content in leaves of each genotype shown in (B). Data are mean ± SEM (n>4). *** *p* < 0.001 (*t*-test).
(TIF)

S4 Fig Kinetic analysis of *CCG* expression in WT and *ein3eil1* with ethylene treatment. Detached third and fourth Leaves from 4-week-old plants were treated with 100 μL/L ethylene for various times. RT-qPCR was performed to quantify the mRNA levels of each gene. *ACT2* was used as an internal control to normalize different samples. The mRNA levels of each corresponding gene in WT at 0 hr were arbitrarily set to 1. Data are mean ± SEM from 3 biological replicates with technical duplicates for each.
(TIF)

S5 Fig Inducible expression of *NYE1* by DEX treatment in *NYE1iox* transgenic line. Four-week-old *NYE1iox* transgenic line was sprayed with 15 μM DEX or 0.05% Methanol (mock) and incubated for two days. The transcript level of *NYE1* in the third and fourth leaves was examined by RT-qPCR. *ACT2* was used as an internal control for normalization. The transcript level of *NYE1* with mock treatment was arbitrarily set to 1. Data are mean ± SEM of 3 biological replicates. ** *p* < 0.01 (*t*-test).
(TIF)

S6 Fig ORE1 promotes *ACS2* expression. qRT-PCR analysis of the *ACS2* transcript levels in third and fourth leaves of 4-week-old WT, *nac2-1*, and *ORE1ox* with 100 μL/L ethylene treatment for 4 d. The *ACS2* transcript level in WT was arbitrarily set to 1. Data are mean ± SEM from 2 biological replicates (one-way ANOVA). Levels not connected by same letter are significantly different. *p* < 0.01.
(TIF)

S7 Fig The phenotypes and chl contents of *nyc1* and *pao* mutants with ethylene treatment. (A) Detached third and fourth rosette leaves from 4-week-old WT, *nyc1*, and *pao* plants treated with 100 μL/L ethylene for 3 d. (B) Quantitative analysis of total chl content in leaves of each genotype shown in (A). Data are mean ± SEM (n = 3). * *p* < 0.05 (*t*-test).
(TIF)

S1 Table Primers used in the study. (XLSX)
