# Supplementary figures and images for: EIN3 and ORE1 Accelerate Degreening during Ethylene-Mediated Leaf Senescence by Directly Activating Chlorophyll Catabolic Genes in Arabidopsis
Source: PLoS Genet. 2015 Jul 28;11(7):e1005399. doi: 10.1371/journal.pgen.1005399 (PMC4517869; doi:10.1371/journal.pgen.1005399)

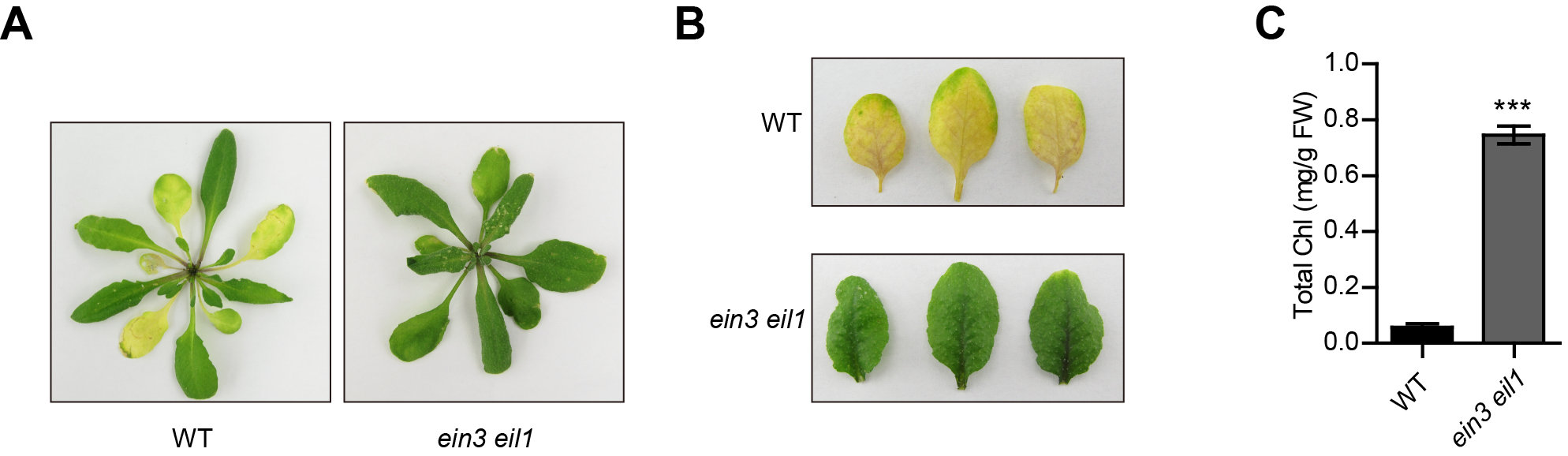

Supplement: S1 Fig — (A) Whole plants of 4-week-old ein3 eil1 mutant showed a stay-green phenotype compared to the wild type (WT) with 100 μL/L ethylene treatment for 4 d. (B) Detached third and fourth rosette leaves from 4-week-old WT and ein3 eil1 plants treated with 100 μL/L ethylene for 4 d. (C) Quantitative analysis of total chl content in leaves of each genotype shown in (B). Data are mean ± SEM (n>4). *** p < 0.001 (t-test). (TIF) [file pgen.1005399.s001.tif]

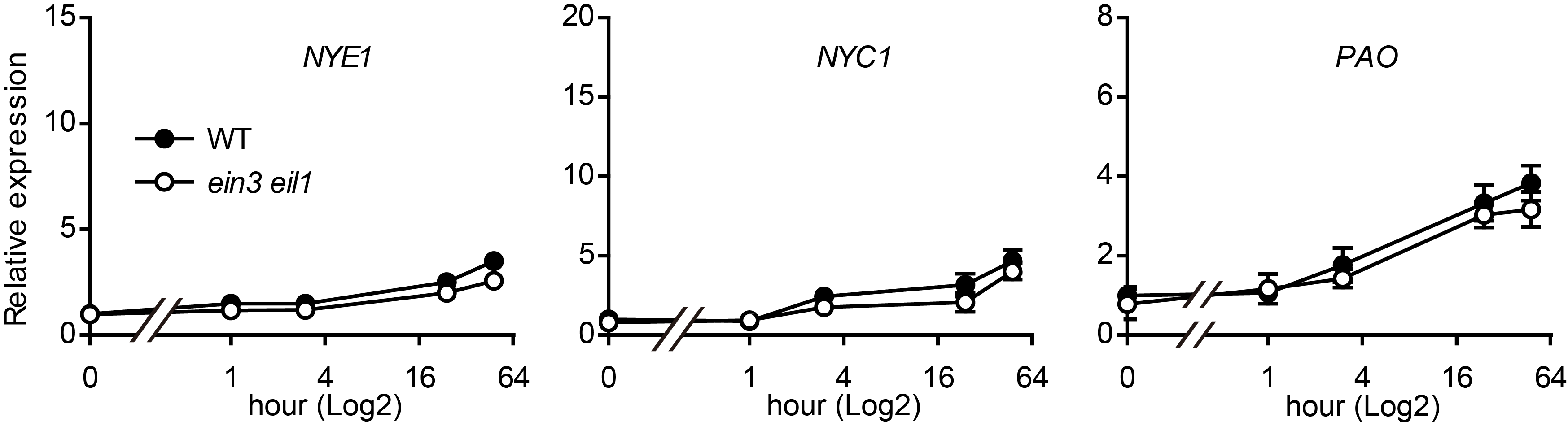

Supplement: S2 Fig — The expression level of each corresponding gene in the WT at 0 hr was set to 1. The scales of y-axes are consistent with that in Fig 2B. Data are mean ± SEM of 3 biological replicates. (TIF) [file pgen.1005399.s002.tif]

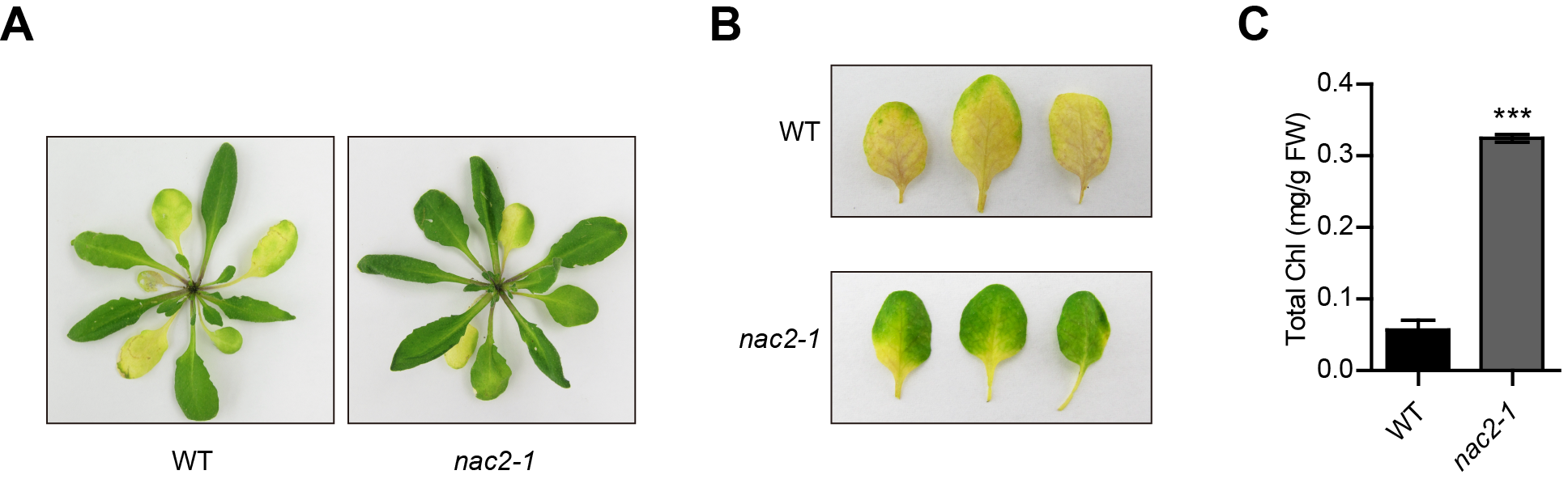

Supplement: S3 Fig — (A) Whole plants of 4-week-old nac2-1 mutant showed a stay-green phenotype compared to the WT with 100 μL/L ethylene treatment for 4 d. (B) Detached third and fourth rosette leaves from 4-week-old WT and nac2-1 plants treated with 100 μL/L ethylene for 4 d. (C) Quantitative analysis of total chl content in leaves of each genotype shown in (B). Data are mean ± SEM (n>4). *** p < 0.001 (t-test). (TIF) [file pgen.1005399.s003.tif]

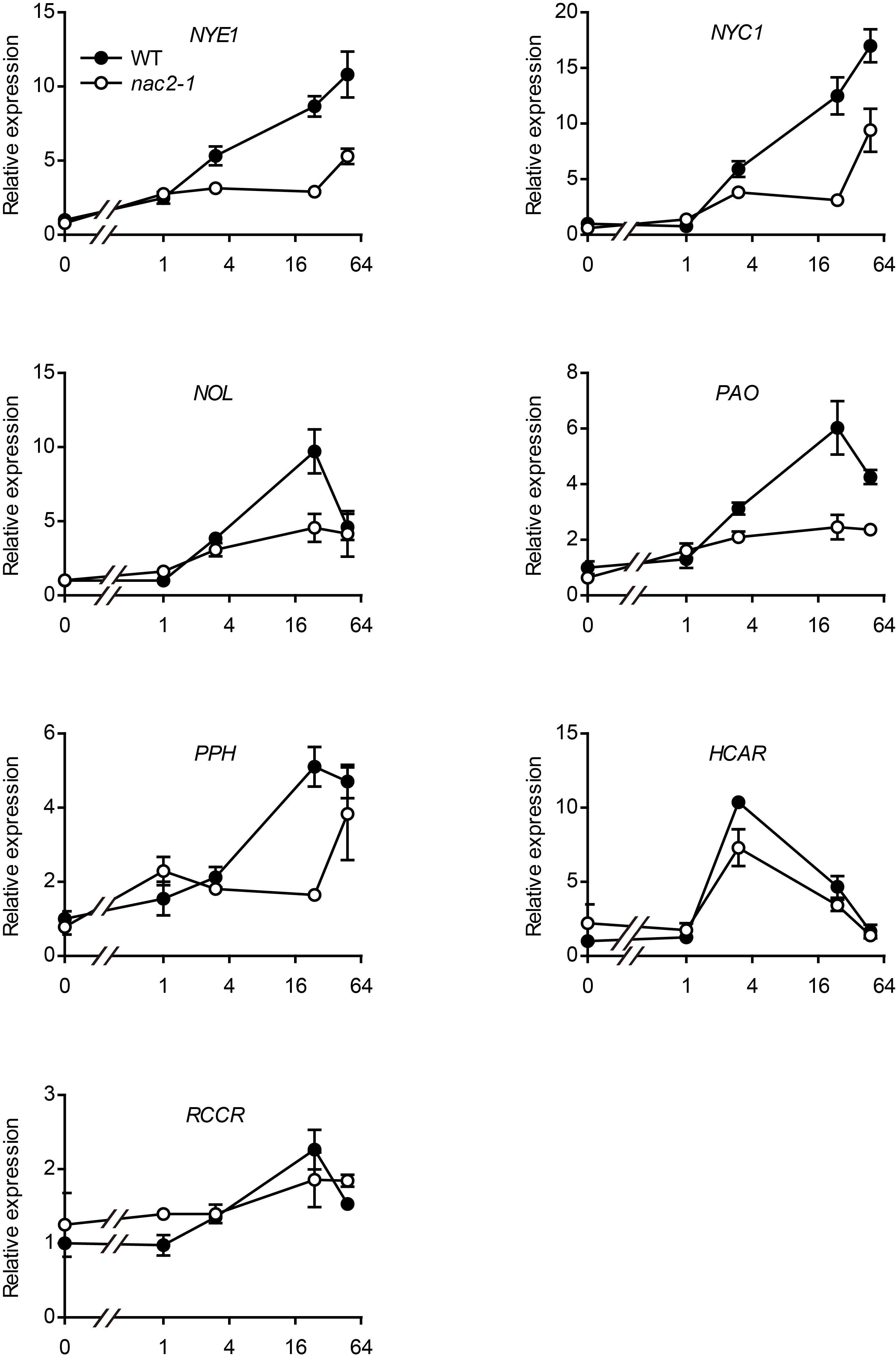

Supplement: S4 Fig — Detached third and fourth Leaves from 4-week-old plants were treated with 100 μL/L ethylene for various times. RT-qPCR was performed to quantify the mRNA levels of each gene. ACT2 was used as an internal control to normalize different samples. The mRNA levels of each corresponding gene in WT at 0 hr were arbitrarily set to 1. Data are mean ± SEM from 3 biological replicates with technical duplicates for each. (TIF) [file pgen.1005399.s004.tif]

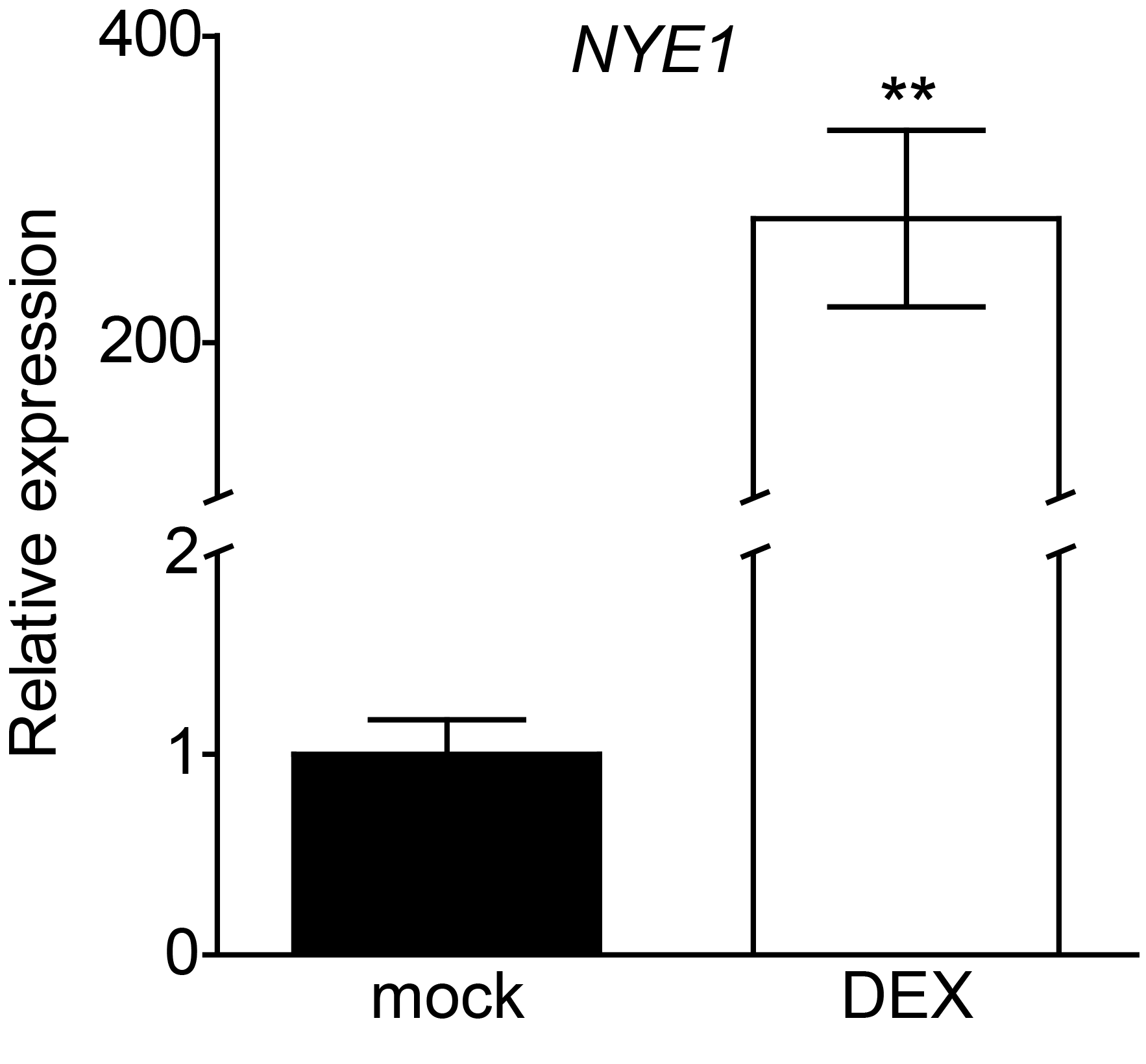

Supplement: S5 Fig — Four-week-old NYE1iox transgenic line was sprayed with 15 μM DEX or 0.05% Methanol (mock) and incubated for two days. The transcript level of NYE1 in the third and fourth leaves was examined by RT-qPCR. ACT2 was used as an internal control for normalization. The transcript level of NYE1 with mock treatment was arbitrarily set to 1. Data are mean ± SEM of 3 biological replicates. ** p < 0.01 (t-test). (TIF) [file pgen.1005399.s005.tif]

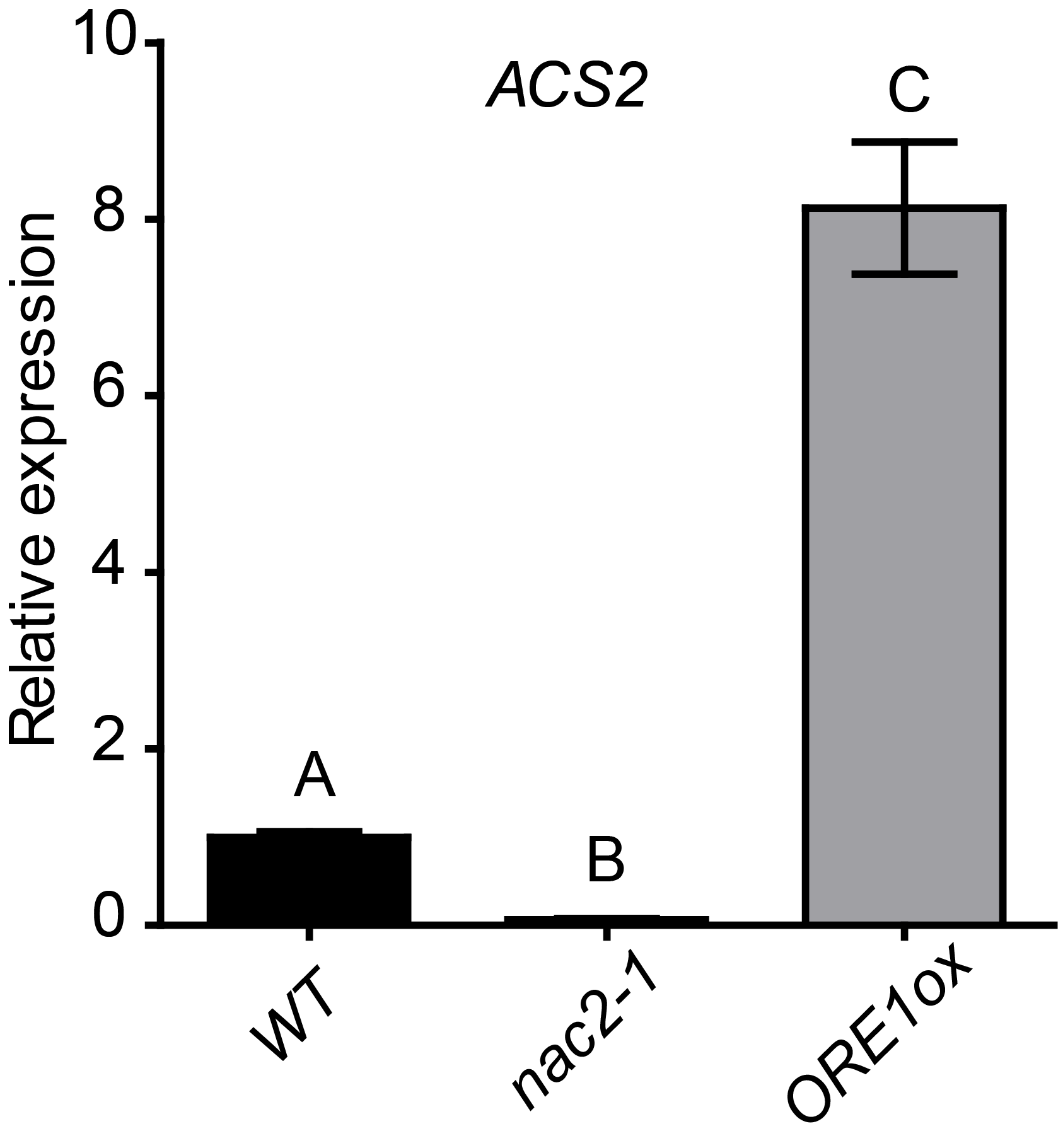

Supplement: S6 Fig — qRT-PCR analysis of the ACS2 transcript levels in third and fourth leaves of 4-week-old WT, nac2-1, and ORE1ox with 100 μL/L ethylene treatment for 4 d. The ACS2 transcript level in WT was arbitrarily set to 1. Data are mean ± SEM from 2 biological replicates (one-way ANOVA). Levels not connected by same letter are significantly different. p < 0.01. (TIF) [file pgen.1005399.s006.tif]

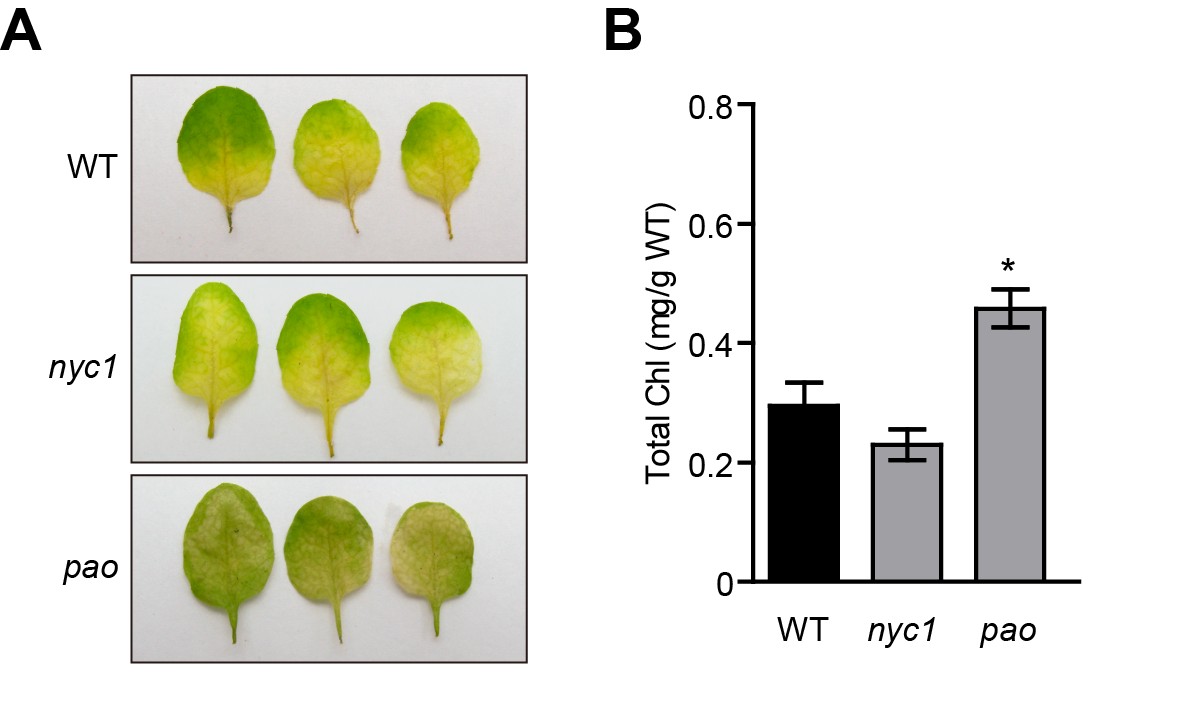

Supplement: S7 Fig — (A) Detached third and fourth rosette leaves from 4-week-old WT, nyc1, and pao plants treated with 100 μL/L ethylene for 3 d. (B) Quantitative analysis of total chl content in leaves of each genotype shown in (A). Data are mean ± SEM (n = 3). * p < 0.05 (t-test). (TIF) [file pgen.1005399.s007.tif]
